# Effect of Dual‐Task Training on Cognitive Function and Physical Condition in Adults With Intellectual Disabilities

**DOI:** 10.1111/jar.70227

**Published:** 2026-04-17

**Authors:** Carmen Gutiérrez‐Cruz, Francisco J. Ruiz‐Perálvarez, Indya del‐Cuerpo, Luis Javier Chirosa‐Ríos, Danica Janicijevic

**Affiliations:** ^1^ Department of Physical Education, Sports and Human Movement Faculty of Teacher Training and Education, Autonomous University of Madrid Madrid Spain; ^2^ Hermanas Hospitalarias, Fundación Purísima Concepción de Granada Granada Spain; ^3^ Department Physical Education and Sports Faculty of Sport Sciences, University of Granada Granada Spain; ^4^ Strength and Conditioning Laboratory, CTS‐642 Research Group, Department Physical Education and Sports Faculty of Sport Sciences, University of Granada Granada Spain; ^5^ Faculty of Sports Science, Ningbo University Ningbo China; ^6^ Department of Radiology Ningbo China; ^7^ Department of Sports Sciences and Physical Conditioning Faculty of Education, Universidad Católica de la Santísima Concepción Concepción Chile

**Keywords:** body composition, choice reaction time, dual task cost, muscle strength

## Abstract

**Background:**

This randomized controlled trial with repeated measures examined the effects of a 21‐week dual‐task training (DTT) program on choice reaction time (CRT), postural control, strength and body composition in individuals with intellectual disabilities.

**Method:**

Fifty‐seven participants with mild to moderate intellectual disability were assigned to an experimental or control group. The experimental group completed 63 training sessions, with evaluations at 12 and 21 weeks, while the control group carried out their regular activities.

**Results:**

A significant reduction in CRT (*p* < 0.005) and improvements in postural control under dual‐task (DT) conditions (*p* ≤ 0.041) were observed with no benefits for DT cost. Mean and maximum strength increased after 12 and 21 weeks (*p* ≤ 0.001). Changes in body composition were characterized by an increase in the percentage of muscle mass (*p* ≤ 0.001) and a reduction in fat mass (*p* = 0.010).

**Conclusions:**

These findings support the effectiveness of DTT for improving CRT and strength in individuals with intellectual disabilities.

## Lay Summary

- This study found that a 21‐week dual‐task training programme—combining physical and cognitive exercises—significantly improved reaction time, postural control, and muscle strength in adults with mild to moderate intellectual disabilities.
- Participants in the program also showed favourable changes in body composition, including increased muscle mass and reduced body fat, which are important for everyday mobility and overall health.
- These improvements suggest that integrated cognitive‐motor training can enhance both physical and cognitive function, which may potentially promote greater independence and employability among individuals with intellectual disabilities
- For the research community, this study provides robust evidence that long‐term, structured dual‐task interventions are effective and feasible in adult populations with intellectual disabilities—a group often underrepresented in intervention studies.

## 1 Introduction

Adults with intellectual disabilities are characterized by an early, multidimensional deterioration that affects both cognitive and motor development (Schalock et al. 2021). Regarding cognitive development, individuals with intellectual disabilities tend to exhibit slower reaction responses and reduced verbal fluency compared to their peers without intellectual disabilities, a disparity linked to cognitive deficits (Affes et al. 2021; Borji, Affes, et al. 2023; Meiran and Shahar 2018). In the context of motor development, individuals with intellectual disabilities exhibit suboptimal performance in terms of parameters related to balance and muscular strength, and concurrently a heightened prevalence of overweight when contrasted with the pears without intellectual disabilities (Blomqvist et al. 2013; Enkelaar et al. 2012; Leyssens et al. 2022; Melville et al. 2007). To a large extent, the lower physical fitness of the adults with intellectual disabilities is related to the impairment of certain cognitive functions and the predominantly sedentary lifestyle that characterize this group (Cleaver et al. 2009; Frey and Chow 2006; Spaniol and Danielsson 2022). All of these factors collectively compromise their ability to perform complex daily activities and pose challenges in securing and maintaining employment independently (Carmeli et al. 2005; Enkelaar et al. 2012; Gutiérrez‐Cruz, Muñoz‐López, et al. 2023). Undoubtedly, enhancing the physical and cognitive abilities of adults with intellectual disabilities increases their prospects in the job market, making them more desirable candidates for employment. In this sense, dual‐task (DT) training programs have been generally accepted as a means for improving cognitive and motor functioning (Bianchini et al. 2022; Brustio et al. 2018; Leone et al. 2017; Borji, Fendri, et al. 2023). These programs involve exercises that require performing two concurrent tasks, typically cognitive and motor. Their assessment is usually done by measuring the changes that occur in DT compared to a single task (ST), which is referred to as the dual‐task cost (DTC). The DTC is based on a competition model between domains, presuming that resources for carrying out a relatively complex DT are limited. Thus, when the demands of concurrent tasks exceed the available resources, the performance in one or both tasks is compromised (Lennie 2003).

Borji et al. (2024) explored the effects of different DT training programs on balance in adults with intellectual disabilities, reporting that DT interference depends on the training modality. Similarly, Pineda et al. (2024) found no conclusive evidence of deficits induced by specific DT conditions in individuals with intellectual disabilities, likely due to the heterogeneity of the study designs used in the studies included in the review. For instance, Mikolajczyk and Jankowicz‐Szymanska (2015a, 2015b) reported that 12 weeks of DT exercises on unstable surfaces resulted in a significant improvement in postural control among adolescents with intellectual disabilities, with the benefits persisting for 8 weeks after the intervention ended. Also, Borji, Fendri, et al. (2023) demonstrated superiority of the DT over ST training for improving cognitive performance and reducing postural sway in adolescents. Similar finding was reported in the group of children with intellectual disabilities, where DT showed its effectiveness in enhancing balance performance (Atak and Algun 2022) and postural control (Kachouri et al. 2022). It is important to note that most of these studies were conducted with children and adolescents as subjects. Considering the known impact of age on postural control performance (Cavalheiro et al. 2009; Huxhold et al. 2006; Phu et al. 2023), it may be questionable to extrapolate these results to adults with intellectual disabilities. Also, all the mentioned interventions did not exceed 12 weeks, which may be insufficient for achieving substantial improvements in highly automated motor tasks that pose significant challenges for individuals with intellectual disabilities. Therefore, the aim of this study was to explore the effects of a 21‐week dual‐task training (DTT) program followed by the group of adults with mild and moderate levels of intellectual disabilities on (I) choice reaction time (CRT), (II) center of pressure (CP) displacement in both ST and DT conditions, (III) static and dynamic manifestations of muscle strength, and (IV) body composition parameters. We hypothesize that DTT will result in (I) a decrease in CRT and (II) reduced CP displacement in both ST and DT conditions. Additionally, we speculate (III) an increase in both static and dynamic strength, accompanied with (IV) favourable changes in body composition, characterized by increased muscle mass and reduced fat mass.

## 2 Methods

### 2.1 Study Design

The present study used a controlled longitudinal pre‐post design with random assignment of the subjects to two parallel groups (one experimental and one control). Their goal was to explore the effects of DTT training program on CRT, postural control parameters, muscle strength, and body composition parameters. The experimental group performed a total of 63 sessions (3 sessions during 21 weeks) of DTT training supervised by the physical education and occupational therapist specialists. To evaluate the process during the implementation of the training program, data collection was performed at three time points and included Pre‐ (Week 1), Mid‐ (Week 13), and Post‐ (Week 24) measurements. These measurements included (I) CRT assessment in a DT condition, (II) postural control parameters evaluation during both ST and DT conditions (sway area, sway length, mediolateral [ML] displacement, and anteroposterior [AP] displacement), (III) muscle strength evaluation (static and dynamic arm and leg force), and (IV) skeletal muscle and body fat mass analysis. The training sessions consisted of three 10‐min exercise blocks and one 20‐min DT walking training block, while the control group continued to perform their usual daily activities in accordance with the regulations of their respective residential care centers. Whenever possible, missed sessions were rescheduled within the same week.

### 2.2 Participants

A priori sample size calculation was conducted using G*Power software (version 3.1.9.6) for a fixed‐design analysis of variance (ANOVA) with time as the within‐subject factor (pre vs. post) and group as the between‐subject factor (control group vs. experimental group) to determine the minimum required sample size. The calculation was based on the CRT, which was selected as the main outcome measure to evaluate cognitive functioning and the effects of the DT training intervention. A small‐to‐moderate effect size (*f* = 0.14) was assumed. This magnitude was chosen conservatively and is consistent with previous studies reporting small to moderate effects of resistance and exercise‐based training programs on cognitive functioning (Cassilhas et al. 2007; Liu‐Ambrose et al. 2010). The analysis used an α level of 0.05, a desired statistical power of 0.80, two groups, two repeated measurements, and an assumed correlation of 0.80 between repeated measures. Based on these parameters, the minimum required total sample size was estimated to be 44 participants.

Initially, 370 individuals from 5 residential and 6 specialized employment centres with mild to moderate intellectual disabilities, sponsored by a Spanish foundation, were selected as the potential sample size. Intellectual disabilities were classified according to the first edition of the WHO International Classification of Diseases and with a score greater than 30% based on the National Government of Spain classification (Royal Decree 1971/December 23, 1999). This classification of intellectual disabilities is a combination of the intelligence quotient level and adaptive behaviour (physical, intellectual, and/or sensory) and is expressed in 5°: nonexistent (0%), borderline (15%–29%), mild (30%–59%), moderate (60%–75%), and severe or profound (≥ 76%). The general inclusion criteria for participation in the research were: (I) have an intellectual disabilities level between 30% and 75% (considered mild to moderate intellectual disabilities), (II) be aged between 18 and 65 years old, (III) not have a motor disability that affects independence or a diagnosed chronic condition that might impact physical performance (individuals with Down syndrome were not included), (IV) not have uncorrected severe vision or hearing problems, and (V) not have lower back pain or lower limb issues that could affect participation in general strength exercises.

**FIGURE 1:**
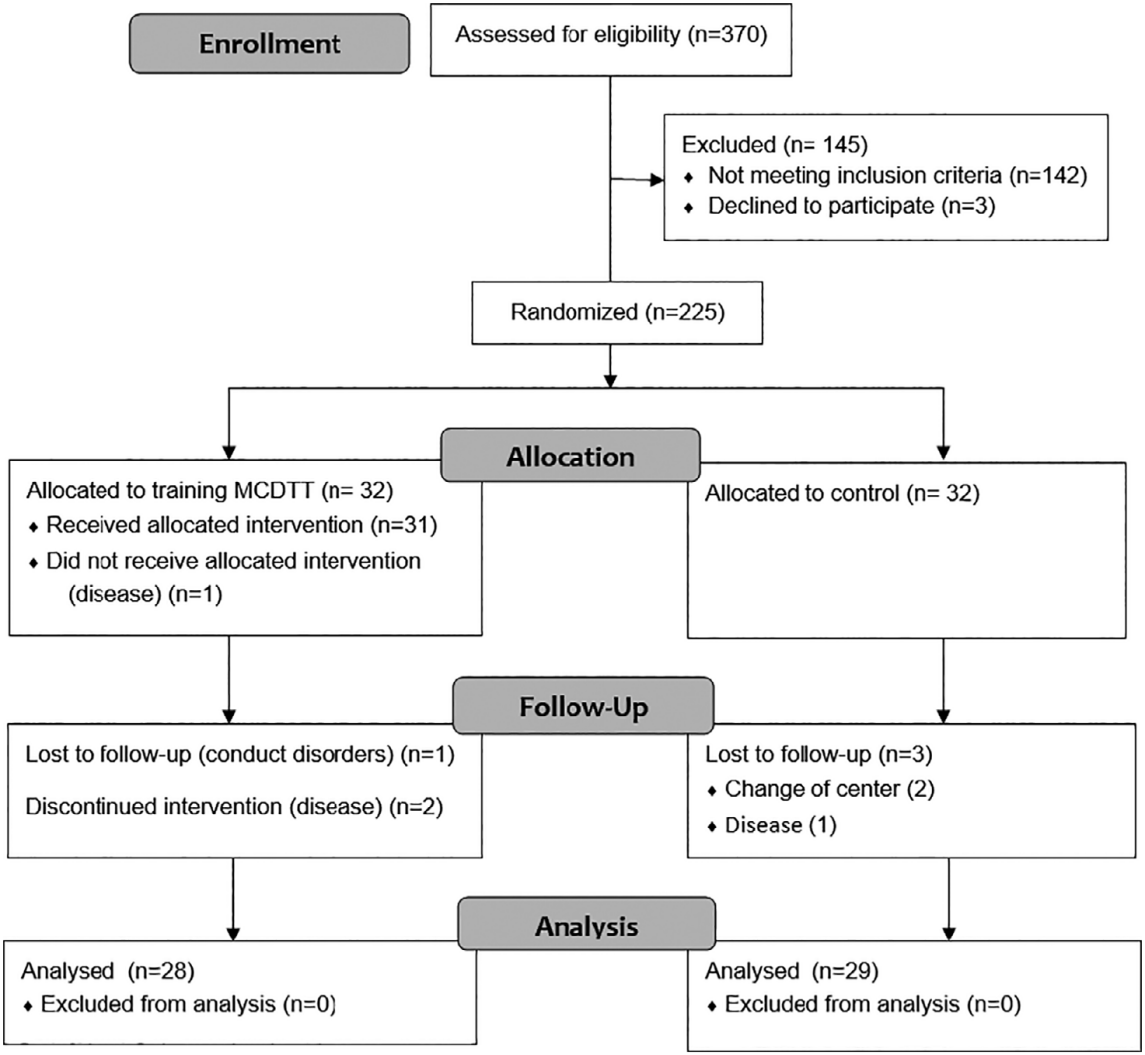
CONSORT flow diagram of participant recruitment, allocation, follow‐up, and analysis. Numbers (*n*) represent the number of participants at each stage.

Out of the 370 people who were initially eligible, 225 met these criteria. Out of 225 participants that met initial criteria, 32 individuals were randomly assigned to the experimental group, and 32 individuals were assigned to the control group. Group assignment was performed using a Merged Block Randomization method (van der Pas 2019), with each block balanced for gender and age to ensure similar characteristics in both groups. Individuals from the experimental group who were unable to complete at least 18 weeks of training or who suffered a serious illness during the training period were not considered for statistical analysis. Thus, during the first week, one participant from the intervention group was excluded due to disruptive behaviours (verbal and physical aggression) when asked to perform the activities of the DTT program. Two additional participants were not included in the statistical analysis due to serious illnesses that prevented them from attending the training sessions for at least 4 weeks. The residential setting facilitated completion of all proposed sessions by the remaining participants. Regarding the control group, two participants were transferred to a Special Employment Center, and another participant experienced a fall prior to the post‐test assessment, which prevented her from completing the post‐intervention measurements. Ultimately, the 28 participants in the experimental group (16 men and 12 women, age = 38 ± 9 years, height = 1.63 ± 0.13 m, weight = 74.6 ± 14.2 kg) and 29 participants in the control group (14 men and 15 women, age = 38 ± 9 years, height = 1.62 ± 0.11 m, weight = 72.0 ± 15.1 kg) completed the DTT training program (see Figure 1 for the CONSORT sample selection flowchart). All selected participants signed the informed consent form themselves or by their legal guardians. The study was conducted in accordance with the Helsinki Declaration standards and was approved by the University's Ethics committee under registration number 2354/CEIH/2021.

### 2.3 DTT

**FIGURE 2:**
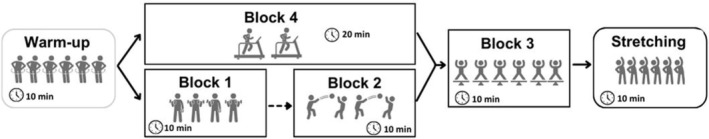
Diagram of the distribution and organization of participants in the training session. Block 1, general dynamic strengthening; Block 2, coordination and throws; Block 3, postural and dynamic balance in dual task; Block 4, gait training in dual task.

The training sessions took place in groups of 5 or 6 people, grouped by age, disability level, and physical condition (i.e., five groups underwent training at different times of the week). Each session began with a standard 10‐min warm‐up consisting of DT‐based gait displacements (e.g., walk according to the coach's instructions, counting backward, saying the names of animals, etc.) and joint mobility exercises (general flexibility exercises). To maintain dual cognitive activity during gait, participants were required to focus their attention and follow the trainer's instructions which were conveyed through pictograms indicating movements such as right, left, backward, and forward. Later on, participants performed the main part of the training which consisted of four blocks. Four out of 6 participants performed blocks 1 and 2, while the remaining two performed the DT gait training (block 4). Subsequently, all six participants performed block 3 and finished with 10 min of active and passive stretching. The order of the participants changed for each weekly session. Thus, each participant completed the warm‐up, block 3 and the final stretching 3 days a week (i.e., in every training session), which was combined with blocks 1 and 2 two days a week, and DT gait training 1 day a week (Figure 2). The pause between the blocks was 3 min, while the detailed description of the blocks is as follows:

#### 2.3.1 Block 1 (General Dynamic Strengthening)

Various exercises were used aiming for the involvement of the largest muscle groups, using body weight and/or external resistances (weighted bars of different masses, elastic bands, battle ropes, and dumbbells). Progress was regulated by increasing the resistance and/or the number of repetitions. Duration of the block was 10 min.

#### 2.3.2 Block 2 (Coordination and Throws)

Participants were instructed to throw wall balls of different masses (< 8 kg) using a variety of movements tailored to individual coordination levels. Progression was regulated by increasing the ball mass, throw distance, and/or the number of repetitions. Duration of the block was 10 min.

#### 2.3.3 Block 3 (Postural and Dynamic Balance in DT)

Participants were instructed to maintain balance on a variety of unstable platforms (e.g., balance board, fitball and bosu). Pictograms (right, left, backward, forward and stop) were used to maintain attention on the task they should perform while maintaining balance (e.g., throwing different types of balls). Exercise intensity was regulated by changing the difficulty level of the tasks while maintaining balance. Duration of the block was 10 min.

#### 2.3.4 Block 4 (Gait Training in DT)

This block consisted of walking at a self‐selected speed on a treadmill that was equipped with a selective attention system placed in front of the participant. The treadmill was connected to a programmable electronic system controlling two sets of three lights each (red, yellow, and green) and a button which allowed CRT recording. The self‐selected speed of walking of each participant was determined in the first session and remained constant for the rest of the sessions. Two levels of cognitive difficulty were applied based on the number of combinations and stimuli presented for a single possible response. During the first 7 training sessions, random lighting of the three lights of a single traffic light was used, while during the last 14 sessions random lighting of the six lights of the two traffic lights was used. Participants were instructed to press the button as quickly as possible when the reaction stimulus (RS) appeared while walking on the treadmill which was a single red light during the first 7 sessions and both red lights during the last 14 sessions. After each session, participants were informed of the average reaction response time and errors made. The duration of the block was 20 min.

To motivate the participants, three reinforcement methods were employed: (I) Verbal persuasion (i.e., coach highlighted individuals' skills and abilities to effectively perform exercises), (II) knowledge of results, provided after each session as experiential reinforcement (displaced mass, number of repetitions, average CRT, and total number of errors in DT gait training); and (III) primary rewards (every 4 weeks, participants who successfully completed all sessions received a meal at a popular restaurant). The small number of participants per group (between 5 and 6) facilitated the administration of these reinforcements, and in no case were they presented as negative reinforcement.

### 2.4 Testing Procedures

All measurements were organized at the Center for Individuals with Intellectual Disabilities before (pre‐week 1), after 12 weeks (mid‐week 13), and after 21 weeks of training (post‐week 24) to collect the following groups of dependent variables:

#### 2.4.1 Postural Assessment and Choice Response Time (CRT)

Participants were asked to stand barefoot in bipedal stance on the Dinascan/IBV force platform (Biomechanical Institute of Valencia, Spain) with arms by their sides, heels separated by 2–3 cm, and feet at a 30°–35° angle. The force platform was equipped with a postural stability analysis system (DedSVE/IBV) which was used to record CP displacement direction and magnitude at 40 Hz. Additionally, the force platform was synchronized with an external system consisting of two traffic lights and a trigger button which was used for CRT measurement (Gutiérrez‐Cruz et al. 2025). Each traffic light consisted of three sets of 25 LEDs, each with a diameter of 0.1 m (red, yellow, and green arranged from top to bottom), which were positioned 3.5 m away from the participant. The trigger button was positioned just in front of participants and at a height corresponding to 70% of each participant's height. An external programmable card allowed for the presentation of various sequences of light stimuli.

After participants assumed correct initial position, they were instructed to remain as still as possible for 20 s under two conditions (I) looking straight ahead (ST) and (II) attending to the light sequence displayed by the two traffic lights, pressing the trigger button as quickly as possible when the two red lights turn on simultaneously (DT). In random order, participants completed a total of 6 valid trials (two in ST and four in DT) with a 2‐min rest between trials. In two out of four valid DT trials, the RS appeared once after the platform data collection ended, and in the other two, the RS randomly occurred twice during platform data collection (these two attempts were considered null for CP records). The CRT was calculated as the difference between the presentation of the RS and the instant the trigger button was pressed. For each valid trial, the CRT in DT condition, and Swing length, Sway area, Mediolateral (ML) displacement, and Anteroposterior (AP) displacement in both ST and DT conditions were recorded. DTC for CP parameters was calculated as the percentage difference between the value obtained during DT and ST conditions using the formula: DTC = [(performance ST − performance DT)/performance ST]·100. The DTC was multiplied by −1 to reflect the inverse relationship between CP trajectory length and postural performance (i.e., a higher DTC reflected a greater decrease in postural balance performance) (Van Biesen et al. 2017). Postural parameters related to the trial with the smallest swing area. For CRT, the highest and lowest values of the four valid recordings under the dual‐task (DT) condition were discarded. Finally, the smallest value was used for both conditions (ST and DT).

#### 2.4.2 Muscle Strength Assessments

**FIGURE 3:**
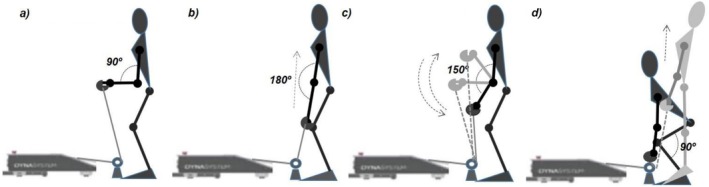
Schematic representation of the muscle strength assessment tests used in the study. Indicated angles represent joint positions and movement ranges during each test. (a) Static arm force, (b) static leg force, (c) dynamic arm force and (d) dynamic leg force.

Static and dynamic strength were recorded using a validated functional electromechanical dynamometer (FEMD) after completing two familiarization sessions (Myo quality, Granada, Spain) (Rodriguez‐Perea et al. 2021; Gutiérrez‐Cruz, Roman‐Espinaco, et al. 2023). The testing session commenced with a 10‐min general warm‐up, followed by the execution of the tests for assessing static arm force, static leg force, dynamic arm force, and dynamic leg force, always in that order (see Figure 3). A 10‐min rest period was implemented between the static and dynamic tests and a 30‐min break between the static leg test and the dynamic arm test. Detailed description of the tests is provided in continuation:

##### 2.4.2.1 Static Strength Tests

The participants assumed an upright position with both arms flexed at 90° (arm test; Figure 3a) and with their knees slightly bent, straight back, and holding a bar with both hands above the knees (leg test; Figure 3b). They were instructed to simultaneously flex both elbows (arm task) or to pull the bar upwards (leg task) with the greatest possible force for 8 s against the immovable resistance imposed by the FEMD system. Three trials were conducted, each separated by a 3‐min rest. For statistical analysis, the mean and maximum force values from the trial recording the highest maximum force were considered.

##### 2.4.2.2 Dynamic Strength Tests

Initial position for the arm task was standing position with elbows flexed at 150° (180° representing full extension), while the initial position for the leg task was having their knees bent at 90°, back straight, and the bar grasped at a height of 0.20 m from the ground. Upon a start signal, participants performed consecutive flexion/extension movements in the elbow (arm task) and lower body joints (leg task) with the maximum range of motion until reaching muscle failure (Figure 3c,d for the dynamic arm and leg force tasks, respectively). For both tests, the FEMD system was programmed to initiate flexion/extension movement with a resistance of 30% of the maximum static force. Resistance increased by 1 kg after each repetition until participants reached muscle failure. Three trials were conducted with 10 min of rest between trials. Data on activity time, total displacement, average force, and maximum force were recorded. Values from the trial with the highest total displacement were used for statistical analysis.

##### 2.4.2.3 Body Composition Assessment

Data pertaining to body fat mass and skeletal muscle mass percentage was gathered utilizing InBody‐230 (Biospace, Seúl, Corea del Sur), which uses the impedance or resistance offered by the body to the passage of two currents with different frequencies (20 and 100 kHz).

### 2.5 Statistical Analysis

Descriptive data are presented as mean ± standard deviation. Normal distribution of dependent variables was verified using the Shapiro–Wilk test (all *p* > 0.062). Homogeneity of variances was tested using the Levene's test, applying Greenhouse–Geisser correction if the assumption of sphericity was violated. A mixed ANOVA was applied with time (Pre vs. Post) as the within‐subject and group (control vs. experimental) as the between‐subject factors. Independent time effect for each group (Pre vs. Post) was determined using Student's *t*‐test for related samples. One‐way repeated measures ANOVA was applied to the three records of the experimental group (Pre, Mid, and Post) with Bonferroni post hoc. Statistical analyses were performed using SPSS software package (IBM SPSS version 22.0, Chicago, IL, USA). Effect sizes were interpreted using eta squared (*η* ^2^), with values of 0.01, 0.06 and 0.14 indicating small, medium, and large effects, respectively (Cohen 1988). Statistical significance was set at *p* ≤ 0.05.

## 3 Results

### 3.1 CRT

**FIGURE 4:**
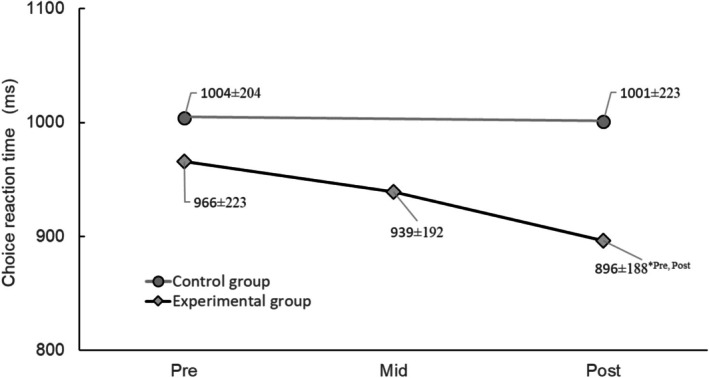
Choice reaction time of the participants belonging to experimental (rhombs) and control groups (circles) before (pre), at the middle (mid) and after training program competition (post). Data are presented as means and standard deviations. *n* = 28 for the experimental group and *n* = 29 for the control group. Results were analysed using a mixed‐design ANOVA. *, statistical significance (*p* = 0.030) compared to pre and mid testing sessions within the experimental group.

Training program reduced CRT in the experimental group, while it remained unchanged in the control group (interaction time × group was significant, *F* (1,42) = 4.967; *p* = 0.030). The main effect of time also reached statistical significance indicating lower CRT at Post measurement (*F* (1,42) = 5.64, *p* = 0.021), which can be attributed to the CRT decrement in the experimental group in the last 12 weeks. Note that the decrement in the first 12 weeks did not reach statistical significance (Figure 4).

### 3.2 Postural Assessment

The intervention was well tolerated, with no reported falls, dizziness, or clinically significant musculoskeletal pain. The interaction time × group was significant for all postural variables during the DT condition and also for the swing length during ST (training program had a decremental effect in the experimental group [positive effect], but not in the control group). The main effect of time never reached statistical significance (all *p* ≥ 0.050). All postural variables except AP displacement during the ST condition (*p* = 0.082) significantly decreased during the training program (*p* ≤ 0.008). No differences were noted between pre and post condition in the control group (all *p* ≥ 0.189) (Table 1).

**TABLE 1:** Results of the mixed‐design analysis of variance (ANOVA) for the pre and post data collection concerning the different variables assessed under single‐task (ST) and dual‐task (DT) conditions.

Variables		ANOVA				Experimental group			Control group		
		Time		Time × group		Pre	Post	*p*	Pre	Post	*p*
		*p*	*η* ^2^_p_	*p*	*η* ^2^_p_						
Swing length (mm)	ST	0.114	0.046	**0.047**	0.071	39.7 ± 13.9	31.5 ± 10.8	**< 0.001**	37.1 ± 16.6	38.1 ± 16.8	0.822
	DT	0.179	0.033	**0.041**	0.075	39.8 ± 11.7	33.5 ± 9.8	**< 0.001**	37.3 ± 14.9	38.7 ± 15.8	0.699
Sway area (mm^2^)	ST	0.441	0.011	0.062	0.063	34.2 ± 27.5	28.6 ± 27.6	**0.047**	31.5 ± 25.7	33.8 ± 27.2	0.470
	DT	0.741	0.002	**0.018**	0.099	43.0 ± 39.0	36.1 ± 34.3	**0.039**	39.7 ± 33.2	44.9 ± 37.7	0.189
ML displacement (mm)	ST	0.249	0.025	0.106	0.048	11.4 ± 5.4	9.6 ± 5.8	**0.008**	11.1 ± 5.8	11.4 ± 5.9	0.789
	DT	0.306	0.019	**0.019**	0.098	13.0 ± 6.8	10.0 ± 5.5	**0.003**	13.3 ± 6.4	14.5 ± 7.7	0.434
AP displacement (mm)	ST	0.050	0.069	0.443	0.011	18.9 ± 8.0	17.0 ± 7.8	0.082	18.5 ± 8.0	17.7 ± 8.3	0.334
	DT	0.246	0.025	**0.035**	0.080	21.6 ± 12.6	18.4 ± 10.0	**0.002**	20.0 ± 7.7	21.0 ± 12.0	0.586

*Note:* Data are presented as mean ± standard deviation for the experimental group (*n* = 28) and control group (*n* = 29). Time (pre vs. post) was treated as the within‐subject factor and group (experimental vs. control) as the between‐subject factor. *p*‐Values correspond to main effects of time and time × group interactions. Statistical significance was set at *p* < 0.05. Bold values indicate statistically significant differences (*p* < 0.05).

Abbreviations: *η* ^2^_p_, partial eta squared; AP, anteroposterior displacement; ML, mediolateral displacement.

When the pre versus post measures of the DTC were compared, all postural variables reached positive values, indicating lower performance for the DT condition compared to ST. DTC was not changed following a DTT program neither in the experimental (*p* ≥ 0.112) nor in the control group (*p* ≥ 0.121). Interaction time × group was not significant for any variable (*p* ≥ 0.138) (Table 2).

**TABLE 2:** Results of the mixed‐design analysis of variance (ANOVA) for dual‐task cost (DTC) variables derived from postural control tests.

Variables	ANOVA				Experimental group			Control group		
	Time		Time × group		Pre	Post	*p*	Pre	Post	*p*
	*p*	*η* ^2^_p_	*p*	*η* ^2^_p_						
Swing length DTC (%)	0.277	0.022	0.381	0.014	3.6 ± 20.5	11.3 ± 28.2	0.135	4.6 ± 25.0	5.4 ± 23.0	0.890
Sway area DTC (%)	0.223	0.027	0.138	0.040	31.1 ± 66.0	29.2 ± 56.7	0.805	37.5 ± 58.9	56.6 ± 74.1	0.121
ML displacement (DTC%)	0.726	0.002	0.395	0.013	17.2 ± 40.1	13.9 ± 43.2	0.704	26.0 ± 43.4	33.9 ± 45.7	0.432
AP displacement (DTC%)	0.645	0.004	0.140	0.040	17.6 ± 42.6	6.4 ± 22.3	0.112	14.3 ± 32.8	20.2 ± 37.9	0.532

*Note:* Data are presented as mean ± standard deviation for the experimental group (*n* = 28) and control group (*n* = 29). Time (pre vs. post) was treated as the within‐subject factor and group (experimental vs. control) as the between‐subject factor. *p*‐Values correspond to main effects of time and time × group interactions. Statistical significance was set at *p* < 0.05.

Abbreviations: *η* ^2^_p_, partial eta squared; AP, anteroposterior displacement; DTC was calculated as: DTC (%) = [(single‐task performance − dual‐task performance)/single‐task performance] × 100; ML, mediolateral displacement.

### 3.3 Muscle Strength

The interaction time × group was significant for all variables (*p* ≤ 0.005) except for activity time during dynamic arm and leg force (*p* = 0.775 and *p* = 0.477, respectively) indicating higher strength improvements in the experimental compared to control group. Main effect of time was significant for all variables except for total displacement during leg force task (*p* = 0.757). The DTT program yielded beneficial effects on 9 out of 12 strength variables, with improvements observed across most metrics except for activity time during dynamic arm and leg force tasks, and total displacement during the dynamic arm force task (Table 3).

**TABLE 3:** Results of the mixed‐design analysis of variance (ANOVA) for the pre and post data collection for the different variables collected during the static and dynamic strength tests.

Variables	ANOVA				Experimental group			Control group		
	Time		Time × group		Pre	Post	*p*	Pre	Post	*p*
	*p*	*η* ^2^_p_	*p*	*η* ^2^_p_						
Static arm force										
Mean force (N)	**0.000**	0.649	**0.000**	0.459	137.0 ± 50.7	227.8 ± 61.4	**0.000**	106.5 ± 52.4	124.0 ± 54.4	0.065
Maximal force (N)	**0.000**	0.507	**0.000**	0.419	174.0 ± 67.3	278.8 ± 78.1	**0.000**	140.4 ± 63.4	149.7 ± 61.1	0.433
Static leg force										
Mean force (N)	**0.000**	0.274	**0.000**	0.287	349.3 ± 109.8	497.8 ± 213.7	**0.000**	316.6 ± 206.1	314.1 ± 162.0	0.934
Maximal force (N)	**0.001**	0.182	**0.000**	0.321	449.5 ± 248.1	617.1 ± 208.8	**0.000**	429.8 ± 241.3	398.4 ± 205.0	0.338
Dynamic arm force										
Activity time (s)	**0.017**	0.098	0.775	0.001	33.48 ± 10.96	30.13 ± 8.21	0.053	34.02 ± 10.92	28.79 ± 11.58	0.126
Total displacement (m)	**0.044**	0.071	**0.000**	0.225	11.9 ± 4.2	12.6 ± 4.4	0.086	12.9 ± 4.8	10.6 ± 3.4	0.002
Mean force (N)	**0.000**	0.559	**0.000**	0.278	93.6 ± 31.5	139.7 ± 39.2	**0.000**	77.6 ± 35.0	91.0 ± 26.5	**0.007**
Maximal force (N)	**0.000**	0.570	**0.000**	0.285	128.6 ± 36.9	196.5 ± 57.0	**0.000**	116.5 ± 33.0	136.3 ± 35.9	**0.002**
Dynamic leg force										
Activity time (s)	**0.001**	1.166	0.477	0.009	41.98 ± 15.56	37.02 ± 9.62	0.050	39.47 ± 15.51	31.82 ± 10.30	**0.015**
Total displacement (m)	0.757	0.002	**0.005**	0.131	8.38 ± 3.65	9.07 ± 3.54	**0.038**	8.10 ± 3.03	7.24 ± 3.04	0.061
Mean force (N)	**0.000**	0.565	**0.000**	0.322	249.0 ± 94.5	405.6 ± 135.6	**0.000**	211.5 ± 92.6	250.1 ± 106.1	**0.021**
Maximal force (N)	**0.000**	0.480	**0.000**	0.501	392.4 ± 150.6	550.3 ± 174.4	**0.000**	366.6 ± 137.0	363.3 ± 143.6	0.854

*Note:* Data are presented as mean ± standard deviation for the experimental group (*n* = 28) and control group (*n* = 29). Time (pre vs. post) was treated as the within‐subject factor and group (experimental vs. control) as the between‐subject factor. *p*‐Values correspond to main effects of time and time × group interactions. Statistical significance was set at *p* < 0.05. Bold values indicate statistically significant differences (*p* < 0.05).

Abbreviation: *η* ^2^_p_, partial eta squared.

### 3.4 Body Composition

**FIGURE 5:**
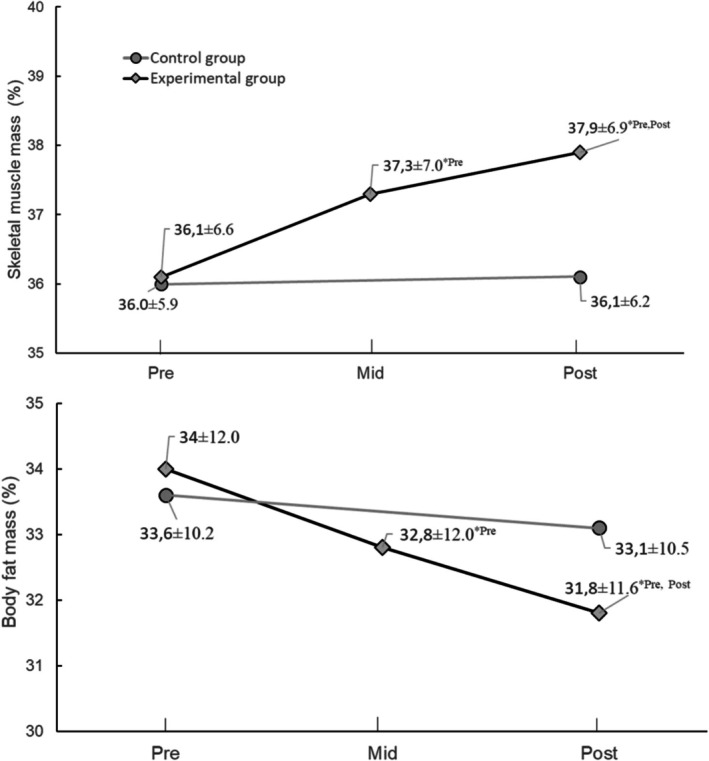
Skeletal muscle mass (top panel) and Body fat mass (bottom panel) of the experimental (rhombs) and control (circles) groups before (pre), at the middle (mid) and after training program competition (post). *n* = 28 for the experimental group and *n* = 29 for the control group. Data are presented as means and standard deviations. Results were analysed using a mixed‐design ANOVA. *p ≤ 0.001 for skeletal muscle mass and *p = 0.010 for body fat mass compared with pre and mid within the experimental group.

The time × group interaction was significant for both skeletal muscle mass (*F* (1, 42) = 15.018, *p* ≤ 0.001) and body fat mass (*F* (1, 42) = 7.129, *p* = 0.010), driven by a notably greater increase in skeletal muscle mass and a more pronounced decrease in body fat mass within the experimental group compared to the control group. Shifts in body composition within the experimental group were marked by a discernible decline in body fat percentage, accompanied by a simultaneous increase in skeletal muscle mass observed at the mid‐testing phase (≈1%). This trend persisted throughout the training sessions, culminating in an approximate 2% alteration at the post‐testing phase. Conversely, the control group exhibited no significant alterations neither in body fat percentage nor in skeletal muscle mass (Figure 5).

## 4 Discussion

The aim of this study was to explore the effectiveness of a DTT with motivational reinforcement on CRT, postural control, static and dynamic strength, and body composition variables in adults with mild to moderate intellectual disabilities. The primary outcomes indicated enhancements in CRT and postural control performance following 21 weeks of DTT training, while improvements in static and dynamic strength, as well as in body composition, were observed both at mid and post measurement sessions (week 13 and 24). These findings hold significance as they suggest the potential for individuals with intellectual disabilities to enhance fundamental life skills essential for autonomous living and successful vocational attainment.

Confirming our first hypothesis, 21 weeks of DTT program reduced CRT of the participants belonging to the experimental group, while it stayed unchanged in the control group. However, no significant reductions in CRT were observed after 12 weeks of training which contrasts with the contributions of Kachouri et al. (2022) and Borji, Fendri, et al. (2023) who reported improvements in the cognitive performance (i.e., verbal fluency) after only 8 weeks of DT training. Furthermore, the verbal fluency test required manipulation of limited information aimed at achieving a specific goal, whereas the CRT test utilized in our study serves as an indicator of the central nervous system's capacity to coordinate movement through the peripheral nervous system (Garg et al. 2013). However, our findings do not contradict the benefits of DT training instead, they corroborate the advantages highlighted in prior studies, albeit necessitating 21 weeks of DT training in this particular investigation. Taken together, these findings suggest that improvements in cognitive outcomes following training may depend not only on intervention duration but also on baseline cognitive functioning and task‐specific cognitive demands. In participants with greater cognitive impairment, longer training periods may be necessary to elicit measurable changes in central processing speed, as reflected by CRT.

The data also showed lower postural performance in the DT condition compared to the ST condition, which occurred both before and after the intervention (Pre and Post) and in both groups (Experimental and Control). Thus, the DTC of all postural parameters remained stable after 21 weeks of DTT training, which is consistent with previous research (Huxhold et al. 2006; Bohlke et al. 2021; Kachouri et al. 2020) and aligns with the general theory on cross‐domain competition, which states that attentional resources are limited (Wickens 2002). Thus, when the attentional demands of two concurrent tasks exceed the available resources, the performance of one or both tasks is compromised. Considering the cognitive and postural impairments present in individuals with intellectual disability, it has been documented that postural balance is more adversely affected compared with their typically developing peers (Gutiérrez‐Cruz et al. 2025).

All postural performance parameters improved in the DT condition after 21 weeks of DTT training, while the control group maintained constant values. These results suggest that DTT training, in addition to improving CRT and motor tasks under DT conditions, facilitated better interaction between the two components (cognitive and motor). However, although to a lesser extent, DTT training also improved postural control performance under ST conditions, which is consistent with previous findings reporting that DT training enhanced postural control in children and adolescents with intellectual disabilities (Mikolajczyk and Jankowicz‐Szymanska 2015a, 2015b; Atak and Algun 2022; Borji, Affes, et al. 2023). Consequently, despite improvements in postural parameters in both ST and DT conditions, the DTT training failed to impact their respective DTCs, suggesting its ineffectiveness in mitigating cognitive‐motor interference during DT compared to ST. The enhanced postural performance during ST may partially owe to the physical conditioning exercises within the program (Borji, Fendri, et al. 2023; Carmeli et al. 2005; Kovačič et al. 2020). However, it is pertinent to note the CRT performance enhancement after 21 weeks, with superior improvements in postural parameters noted in the DT condition compared to ST, suggesting a potential role of the cognitive component in augmenting postural balance during DT. This pattern supports the notion that DT training may preferentially enhance postural control when attentional resources are concurrently challenged. These results resonate with earlier studies indicating the explanatory role of combined physical and cognitive training in enhancing postural control (Atak and Algun 2022; Borji, Fendri, et al. 2023).

The effects of DTT in individuals with intellectual disability may be explained through different complementary theoretical frameworks, including the theory on cross‐domain competition and the task automation hypothesis, which suggests that extensive practice allows tasks to become automated, thereby reducing their attentional demands and minimizing interference under dual‐task conditions (Ruthruff et al. 2001). Thus, it can be speculated that the practice of balance exercises included in the DTT program may have induced a certain degree of automatization of postural tasks, consequently decreasing the attentional demands required for their execution and allowing greater attentional capacity to be allocated to the concurrent cognitive task, thus bringing the training closer to real‐life social demands.

As previously stated, improvements in postural balance might be partly explained by the physical conditioning training implemented in the DTT. In this regard, several studies have reported that increased strength and reduced body mass enhance postural control performance (Handrigan et al. 2010; Kachouri et al. 2016; Wiacek et al. 2009). Given the significant increase in both static and dynamic strength and the reduction in body mass reported for the experimental group, this could provide a coherent explanation for the results. However, caution must be exercised when attributing the enhancement of postural control solely to increased strength or certain body composition factors. For instance, Granacher and Gollhofer (2012) and Gutiérrez‐Cruz, Roman‐Espinaco, et al. (2023) found no correlation between static and dynamic strength and postural control performance in groups of adolescents and adults with intellectual disabilities, Blomqvist et al. (2013) reported only minor correlations between body mass index and strength with balance, while Gobbo et al. (2014) found no evidence that physical activity leads to clear benefits in improving static or dynamic balance in a DT condition. These findings indicate that DT training improves the quality of dynamic force execution, which may be relevant for functional movements performed under cognitively demanding conditions.

The data also affirm our third hypothesis, demonstrating that the experimental group experienced notable increases in both mean and peak strength following 12 and 21 weeks of DTT training compared to the control group. These enhancements spanned across assessments evaluating both static and dynamic strength. Furthermore, there was a noticeable rise in total displacement during dynamic arm and leg tests, while no alterations were observed in activity time. Consequently, the DTT training proved effective in augmenting the execution speed of dynamic tests, achieved at a relatively higher resistance, as the protocol mandated an initial resistance set at 30% of maximum static force. In conjunction with strength gains, the DTT intervention also demonstrated effectiveness in reducing fat mass and enhancing muscle mass among adults with intellectual disabilities, evident after 12 and 21 weeks of intervention.

The primary strength of this study lies in its successful implementation of an intensive intervention protocol for individuals with intellectual disabilities, consisting of three sessions per week over a span of 21 weeks (involving a total of 57 participants). Notably, the research team demonstrated commendable effort in navigating the challenges inherent in executing the training program for individuals with limited interest and motivation toward exercise (Lee et al. 2016). Emphasizing the role of motivational reinforcements integrated into the DTT training is crucial for understanding the intervention's success. However, certain limitations warrant acknowledgment. Firstly, the lack of proposed procedures that would allow CTR recording in the ST condition without the initial position interfering with the response time. Secondly, the age range of our participants was very heterogeneous (18 to 65 years old). Third, the study included multiple outcome variables across different functional domains. Although post hoc comparisons were adjusted using Bonferroni correction, no additional global correction for multiple testing was applied. Fourthly, although the program appeared effective, the absence of an active control group limits internal validity and makes it impossible to determine whether the observed benefits were specific to the dual‐task component or could also be achieved with comparable non‐DTT programs. Fifthly, although the cognitive and strength measures used are widely applied, potential ceiling or floor effects cannot be ruled out and may have influenced the sensitivity to detect changes in some participants, particularly given that most participants were low to moderately active in their residential settings. Lastly, the complex cognitive and motor impairments in individuals with intellectual disabilities present challenges in uniformly assigning cognitive tasks with similar difficulty levels. For future studies, it is recommended to limit the range of intellectual disabilities among the participants assigned to the studies.

## 5 Conclusions

The DTT program, together with the motivational reinforcements included, elicited positive effects on all dependent variables among participants in the experimental group, contrasting with the control group maintaining their routine daily activities without observable changes. Specifically, upon completion of the DTT program, the experimental group displayed reduced CRT and enhanced postural performance in the DT condition. However, despite some improvement, the ST condition showed a comparatively weaker enhancement, signifying that DTT training did not diminish the DTC, thereby sustaining cognitive‐motor interferences in the DT versus ST condition. Furthermore, the experimental group exhibited notably superior enhancements in both static and dynamic strength compared to the control group. This was complemented by an increased distance covered in dynamic assessments, while maintaining consistent execution time, indicative of heightened execution speed. The DTT training also proved effective in reducing fat mass and augmenting skeletal muscle mass. The amplified strength and favourable alterations in body composition might contribute to the enhanced postural control observed. In summary, these findings underscore the efficacy of a 21‐week DTT regimen in enhancing CRT, postural control, strength, and body composition among individuals with intellectual disabilities. Future studies should consider limiting the age range and level of intellectual disability of included participants, as well as incorporating an active control group to allow determination of whether DTT produces effects comparable to or superior to those of a general recreational program of the same duration without a dual‐task component.

## Author Contributions

All authors have read and approved the final version of the manuscript.

## Funding

UGR‐VivaGym‐Fundación Adecco Research Chair for the Labor Integration of People with Disabilities through Sports (Code: 01/22 ENPINCLADI).

## Ethics Statement

The study was conducted in accordance with the Helsinki Declaration standards and was approved by the university's ethics committee (No. 2354/CEIH/2021).

## Consent

All selected participants signed the informed consent form themselves or by their legal guardians.

## Conflicts of Interest

The authors declare no conflicts of interest.

## Data Availability

The data that support the findings of this study are available on request from the corresponding author. The data are not publicly available due to privacy or ethical restrictions.
